# A comparative analysis of weekly internal and external workloads between starting and non-starting professional soccer players: A retrospective 21-week examination

**DOI:** 10.1371/journal.pone.0309475

**Published:** 2024-08-28

**Authors:** Philipp Kunz, Peter Düking, Billy Sperlich

**Affiliations:** 1 Integrative and Experimental Training Science, Institute of Sport Sciences, University of Würzburg, Würzburg, Germany; 2 Department of Sports Science and Movement Pedagogy, Technische Universität Braunschweig, Braunschweig, Germany; Universidad Autonoma de Chihuahua, MEXICO

## Abstract

**Objectives:**

Aims of the present investigation encompassed: (i) the quantification of training and match loads experienced by starters and non-starters within a professional soccer team; (ii) the identification of variations in these loads across different training modalities, namely, Preparation Training (PT), Match and Match Compensation Training (M&MCT), and their cumulative sum (Total Load; TL); and (iii) the formulation of guidelines aimed at harmonizing the weekly workloads between the groups.

**Methods:**

Internal and external load of training sessions (n = 97), competitive matches (n = 21) and running based sessions (n = 4) were recorded for 21 weeks to investigate possible heterogeneity between starters and non-starters across PT, M&MCT and TL.

**Results:**

During PT, time spent in heart rate zone 5 (HRZ5) was increased for non-starters (+46.1%). During M&MCT, lower loads for non-starters were found in the rate of perceived exertion (sRPE) (-45.6%), HRZ4 (-54%) and HRZ5 (-77.8%), total distance (TD) (-37%), number of sprints (-58.1%), distance in speed zone 1 (-51.1%), zone 3 (-61.5%) and zone 4 (-59.8%) (SZ1, SZ3&4) and all acceleration and deceleration zones (Acc1-4; Dec1-4) (Acc1: -53.1%; Acc2: -56.3%; Acc3: -59.2%; Acc4: 57.8%) (Dec1: -45.9%; Dec2: -55.2%; Dec3: -63.2%; Dec4: -67.7%). Regarding TL, the non-starters’ loads remain lower compared to starters for sRPE (-19.2%), HRZ4 (-21.6%) and HRZ5 (-41.4%), number of sprints (-26.7%), SZ3 (-34.2%), Acc3 (-24.4%), Acc4 (-26.1%), Dec2 (-18.7%), Dec3 (-24%) and Dec4 (-31.2%).

**Conclusions:**

By implementing a running-based regimen on matchday and MCT the day after (MD+1), TD, distances in SZ1, SZ2, SZ4, SZ5, and counts of accelerations in Acc1&2, as well as Dec1, were effectively replicated for non-starters. All other variables remained unaligned for the non-starters. Given the prevalent emphasis on Small-Sided Games (SSGs) during MCT at MD+1, the incorporation of an additional running-based session for non-starters on MD is advised to address gaps in TD, sprint counts, and high-intensity load variables, such as HRZ4&5 and SZ 3 to 5.

## Introduction

The nature of professional soccer matches, typically consisting of two 45-minute halves, imposes substantial metabolic, cardiovascular, neuromuscular, and psychological demands on the starting players playing for most of the 90 minutes. Quantitative analyses of external load metrics indicate that players cover a total distance ranging from 10 to 13 km [1–5] including approximately 25% of runs executed at high intensity (>14.4 km/h), 8% at very high intensity (>25.2 km/h), along with approximately 70 to 80 accelerations (> 2 m**/**s^2^) and 55 to 85 decelerations (< -2 m**/**s^2^) [6, 7]. With respect to internal load variables, players experience an average of 85–91% of their maximum heart rate (HR_max_) during matches [8–12]. Given these workload characteristics, competitive matches constitute the most potent stimulus within the weekly conditioning regimen for soccer athletes [13].

In view of the increased numbers of competitive demands [14], it becomes essential to ensure an equitable distribution of match-related workloads throughout the entire team, driven by two principal considerations: i) to sustain sport-specific fitness levels for all players, and ii) to mitigate the risk of injuries stemming from either chronic or acute overload, as players availability is associated with higher chances of success [15]. The practice of consistently fielding the same starting lineup can result in excessive physiological and psychological stress for those players, concomitantly elevating their injury risk [16]. Simultaneously, this approach would yield a diminished workload and preparedness for substitute players, who accumulate significantly less actual match-play time. One methodological approach to individually quantify external workload and assess both acute and chronic stress levels for each player—thereby addressing the inherent workload heterogeneity within the team—is the utilization of Global Navigation Satellite Systems (GNSS). These systems facilitate the monitoring of running-based metrics, such as total distance covered, high-intensity running, and the frequency of accelerative and decelerative movements [17, 18].

To address the issue of workload disparity between starting and non-starting players, a prevalent strategy involves subjecting non-starters to running-based sessions on Match Day (MD) and/or supplementary training sessions on the day following the match (MD+1) to equilibrate their weekly load with that of the starters. However, the cohort of non-starters typically consists of a limited number of players, constraining the organizational feasibility of employing soccer-specific training modalities requiring larger participant numbers for match compensation training (MCT).

In this context, Small-Sided Games (SSGs) are frequently employed as a surrogate measure to emulate the technical, tactical, and physiological aspects of a match. However, SSGs are limited to a smaller pitch size in relation to the number of players. Therefore, during SSGs players are exposed to less total distance [19], running distance in higher speed zones (>19.8 km/h) [19], number of accelerations [20] and maximal sprinting speed [19, 21] when compared to match conditions. Conversely, SSGs yield comparable or even elevated average HR responses, contingent upon player count and pitch dimensions, as well as a similar proportion of time spent in very high-intensity zones (>95% HR_max_) [8].

However the total time spent in high heart rate zones during official matches (i.e. approximately 80–90% HR_max_ for 90 minutes) [22] is not adequately replicated in SSGs due to their substantial shorter duration (i.e. 4x4min intervals). This highlights the significance of the high-intensity domain when contrasting starters and non-starters [23].

Given the cumulative deficit in both internal and external loads for non-starters relative to starters, coaching staff must scrutinize the long-term physical development of non-starters, who may be inadequately prepared for match demands. To the best of our knowledge, there is currently no existing dataset that examines the internal and external workloads of both starting and non-starting players within a professional soccer team over an extended duration.

Based on existing literature and practical experience, we hypothesized that there would be a balanced internal and external load between starters and non-starters during preparation training, but a reduced workload for non-starters during Match and Match Compensation Training (M&MCT). The extent of the discrepancy between the loads during Preparation Training (PT) and (M&MCT) is still unclear, necessitating further investigation to develop practical recommendations.

Therefore, the objectives of this study were: i) to quantify the internal and external loads of both starting and non-starting players over a 21-week period, ii) to compare workloads during weekly PT, M&MCT, and the aggregated workload of PT and M&MCT (total load; TL), and iii) to offer empirically-based practical recommendations to mitigate workload heterogeneity between starters and non-starters within the team.

## Materials and methods

### Data collection procedure

Data pertaining to all outdoor training sessions and competitive matches were systematically collected during the 2019/2020 season, spanning from September 9, 2019 to March 8, 2020. This dataset encompasses 21 competitive matches, 97 unified training sessions (involving both starters and non-starters), four standardized running-based sessions conducted at home, and 17 segregated training sessions (differentiating between starters and non-starters). Weekly gym-based sessions, which were not monitored, are excluded from the scope of this study. In addition, all data collected from activities outside of routine team training, such as individual sessions or rehabilitation training for specific players, were excluded from the analysis. As all calculations are based on a group level, no individual load data could be assigned to the players.

**Fig 1:**
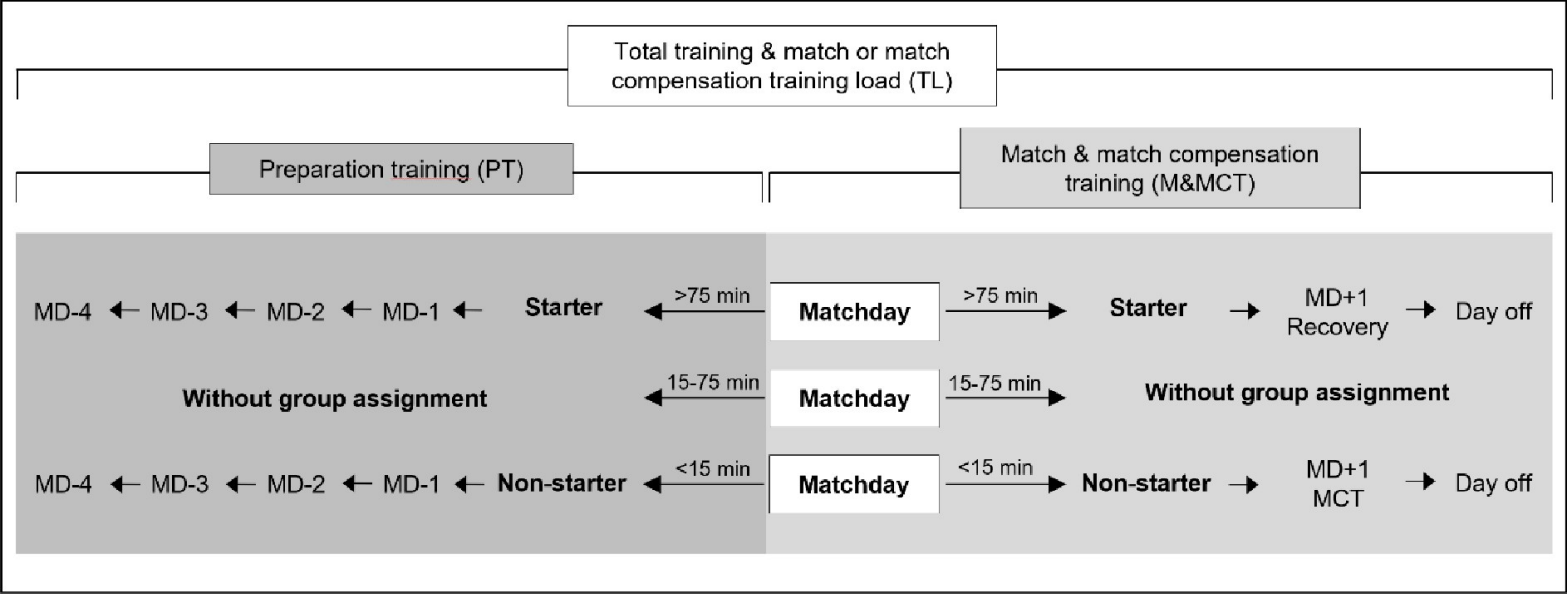
Exemplary microcycle for the division into groups of starters and non-starters based on the playing time. MCT = Match compensation training.

For data analysis, players were categorized into distinct groups based on their projected playing time in the upcoming competitive match. The analysis of soccer matches is often carried out by dividing the full length into smaller segments, frequently applied are 3x15 minutes per half [24–26]. Specifically, the team was bifurcated into starters (with playing time exceeding 75 minutes in the corresponding match) and non-starters (with playing time less than 15 minutes). All players who did not fall into either the starter or non-starter group (i.e., those with a playing time of 15–75 minutes) were excluded from the data analysis for the respective week. This categorization informed the structuring of the entire training week associated with the forthcoming match, denoted as PT (Fig 1). This also included subsequent training sessions designed to recalibrate the internal and external workloads of non-starters (occurring on MD+1 and MD+2), collectively termed as MCT. Depending on the number of players available, SSGs with adjusted field dimensions formed the majority of the load in MCT. Dimensions were based on previous studies, ranging from 20x30m and 25x35m to 30x40m for 4vs4, 5vs5 and 6vs6 respectively [27].

Additionally, running-based sessions were conducted on the match day for players not included in the squad, as well as post-match runs for players with limited playing time (less than 45 minutes) (see Fig 1). Recovery runs were also scheduled for starters with substantial playing time (greater than 45 minutes) on MD+1 (see Fig 1).

The combined regimen of PT and M&MCT was designated as a single weekly microcycle. Given a total player count of 28 and 21 matches, the study analyzed 588 datasets. Of these, 32% (n = 190) pertained to starters, 61% (n = 356) to non-starters, while 7% (n = 42) were excluded from evaluation due to intermediate playing times ranging between 15 and 75 min. Weeks with no competitive match (n = 4) as well as the weeks during the winter preparation phase (n = 2) were not included in this evaluation.

### Subjects

The external and internal load data of twenty-eight professional soccer players (goalkeepers excluded) from a professional German soccer team (age: 23.4 ± 3.5 years; body height: 184 ± 7 cm; body mass: 77.5 ± 8 kg; experience on adult professional level: 4.9 ± 3.7 years) were investigated in this study. Normal training weeks consisted of 5–6 training sessions, with variation depending on the match schedule. During the period under review, only one match took place per week. All participants gave their written informed consent. As all data used in the current study were collected through the daily monitoring routine of the club, no ethical approval was required [28].

### Measurements

Various indices of physical activity, encompassing heart rate (HR) and locomotor parameters, were systematically monitored during each on-field training session, competitive match, and running-based session utilizing a portable Global Positioning System (GPS) operating at a 10Hz frequency (Polar Team Pro, Polar, Kempele, Finland). The reliability and accuracy of this monitoring system have been validated elsewhere [29]. Each player was equipped with a single device, affixed to a chest strap, which was worn 15 minutes prior to and 15 minutes following each session. The data subsequently analyzed were confined to the active duration of each training session, commencing with the warm-up and concluding with the final team drill.

Internal outcome variables included the session rating of perceived exertion (sRPE) and the time allocated across individual heart rate zones 1 to 5 (HRZ 1–5) (refer to Table 1). The sRPE, gauged using the Borg CR-10 scale [30], was ascertained 30 minutes post-session by querying each player’s level of physical exertion, with a scale ranging from 1 (indicative of minimal exertion) to 10 (indicative of maximal exertion) on an online survey on Google Forms (Google LLC, Mountain View, United States of America). HR zones were computed based on each player’s maximum heart rate (HR_max_), which was determined via a shuttle run test (Yo-Yo Intermittent Recovery Level 2), as previously described by Krustrup et al. [31].

**Table 1:** Overview of the defined zones for heart rate and running variables.

Zones	% of maximum heart rate [%]	Running Speed [km·h^-1^]	Acceleration [m·s^-2^]	Deceleration [m·s^-2^]
1	50–60	3.00–6.99	0.50–0.99	-0.50 –-0.99
2	60–70	7.00–10.99	1.00–1.99	-1.00 –-1.99
3	70–80	11.00–14.99	2.00–2.99	-2.00 –-2.99
4	80–90	15.00–18.99	> 3.00	> -3.00
5	90–100	> 19.00		

External outcome variables subjected to analysis encompassed the total distance covered (TD), the number of sprints (SP) (characterized by accelerations exceeding 2.8 m/s^-2^), the distance traversed within five distinct speed zones (SZ 1–5) (measured in meters) (refer to Table 1), and the counts of accelerations (Acc 1–4) and decelerations (Dec 1–4) distributed across four respective zones (refer to Table 1).

### Statistics

Data are presented as mean ± standard deviation, accompanied by a 95% confidence interval (CI) for each player cohort across all variables. The normality of the data distributions was assessed utilizing the Kolmogorov-Smirnov test. To evaluate differences between starters and non-starters, an independent t-test was employed across the three distinct evaluation segments—PT, M&MCT, and TL—with a predetermined significance threshold set at *p* < 0.05.

Additionally, Cohen’s *d* effect size was determined using a pooled SD [32] for all variables of PT, M&MCT and TL with the following interpretation: < 0.20 = *trivial*, 0.2–0.59 = *small*, 0.6–1.19 = *moderate*, 1.20–1.99 = *large*, 2.00–3.99 = *very large* and > 4.00 = *extremely large* effect [33].

Statistical analyses were performed using GraphPad Prism Version 10.0.2 for Windows (GraphPad Software, San Diego, California).

## Results

### Distribution of grouping and match playing time within the team

Among the 28 players, a subset of 10 players who were most frequently designated as starters contributed to 82.1% of all data for this group, accounting for 156 out of 190 possible allocations to the starter category. These players cumulatively logged 14,982 minutes in matches, averaging 71.3 ± 11.4 minutes per player per match, thereby constituting 79.3% of the total match-playing time. Conversely, another subset of 10 players received no allocations to the starter group and collectively accrued 207 minutes of match-playing time, which represents 1.1% of the overall playing time and averages to 1.0 ± 1.9 minutes per player per match.

### Internal and external load of all sessions for M&MCT

Mean internal and external loads of all possible sessions for M&MCT are presented in Table 2.

**Table 2:** Overview of the mean internal and external load for all possible sessions for M&MCT.

	Mean internal and external loads at M&MCT for non-starters		Mean internal and external loads at M&MCT for starters	
Variables	Run for non-starters (MD) Mean ± SD (95% CI)	MCT (MD+1) Mean ± SD (95% CI)	Match (MD) Mean ± SD (95% CI)	Recovery run for starters (MD+1) Mean ± SD (95% CI)
**Internal Load Variables**				
sRPE (AU)	246.9 ± 21.8 (214.0–279.8)	439.9 ± 130.7 (232.0–647.8)	1002.2 ± 73 (898–1106.5)	75.6 ± 12.2 (55.8–95.4)
HRZ1 (min)	6.1 ± 1.7 (3.5–8.6)	11.7 ± 4.7 (4.2–19.1)	9 ± 2.8 (5–13)	4.6 ± 1.6 (1.9–7.3)
HRZ2 (min)	7.6 ± 1.1 (5.9–9.3)	16.6 ± 3.3 (11.3–21.8)	9 ± 1.1 (7.4–10.6)	12.9 ± 1.4 (10.6–15.2)
HRZ3 (min)	7.5 ± 1.2 (5.7–9.3)	13.7 ± 3.5 (8.1–19.3)	16.1 ± 7.8 (5–27.1)	9.6 ± 1.4 (7.3–11.9)
HRZ4 (min)	8.9 ± 1.2 (7.2–10.7)	13.9 ± 3.3 (8.6–19.2)	39.4 ± 3.7 (34–44.7)	1.7 ± 0.6 (0.7–2.7)
HRZ5 (min)	0.6 ± 0.4 (0–1.3)	9.6 ± 3.9 (3.4–15.8)	33.8 ± 6.7 (24.2–43.4)	-
**External Load Variables**				
TD (m)	4255.5 ± 146.5 (4034.9–4476.2)	5633.7 ± 1408.5 (3392.8–7874.6)	10713.0 ± 277.1 (10317.4–11108.5)	4396.8 ± 99.9 (4235.0–4558.6)
Sprints (n)	-	13.2 ± 8.7 (-0.7–27.0)	28.5 ± 5 (21.4–35.6)	-
SZ1 (m)	272.5 ± 28.0 (230.3–314.7)	1697.3 ± 633.5 (689.5–2705.2)	3391.3 ± 183.8 (3128.9–3653.7)	88.2 ± 17.5 (59.8–116.6)
SZ2 (m)	2576.3 ± 150.1 (2350.2–2802.3)	1906.1 ± 738.7 (730.9–3081.3)	2446.8 ± 130.7 (2260.3–2633.3)	2115.5 ± 129.2 (1906.2–2324.8)
SZ3 (m)	261.1 ± 22.9 (226.7–295.6)	986.3 ± 707.1 (-138.7–2111.3)	2316.8 ± 176.6 (2064.7–2568.9)	2165.1 ± 120.9 (1969.3–2360.9)
SZ4 (m)	153.1 ± 14.8 (130.8–175.4)	556.8 ± 641.2 (-463.4–1576.9)	1224.0 ± 127.7 (1041.7–1406.3)	2.9 ± 2.7 (-1.4–7.2)
SZ5 (m)	885.2 ± 34.5 (833.2–937.2)	186.9 ± 145.3 (-44.2–418.1)	878.9 ± 84.4 (758.4–999.4)	-
Acc1 (n)	29.4 ± 5.1 (21.8–37.0)	235.4 ± 101.6 (73.6–397.1)	427.3 ± 41.5 (368.1–486.6)	27.0 ± 4.6 (19.6–34.4)
Acc2 (n)	22.8 ± 2.5 (19.1–26.5)	136.8 ± 62.4 (37.5–236.1)	284.0 ± 66.3 (189.4–378.6)	3.4 ± 1.8 (0.5–6.3)
Acc3 (n)	1.9 ± 1.1 (0.3–3.6)	41.8 ± 22.7 (5.8–77.9)	89.1 ± 11.1 (73.3–104.9)	-
Acc4 (n)	-	9.6 ± 6.9 (-1.3–20.6)	20.3 ± 4.1 (14.5–26.2)	-
Dec1 (n)	51.3 ± 4.3 (44.9–57.8)	285.5 ± 109.4 (84.5–432.4)	445.0 ± 38.6 (389.9–500.2)	28.3 ± 5.8 (18.9–37.7)
Dec2 (n)	28.4 ± 3.4 (23.3–33.5)	150.0 ± 68.4 (41.1–258.9)	303.3 ± 37 (250.5–356.2)	7.0 ± 1.3 (4.8–9.2)
Dec3 (n)	1.9 ± 1 (0.4–3.5)	36.1 ± 19.3 (5.3–66.9)	83.7 ± 8.3 (71.9–95.6)	-
Dec4 (n)	-	10.7 ± 7.3 (-0.9–22.4)	28.7 ± 3.6 (23.5–33.9)	-

sRPE, session rate of perceived exertion; HRZ1-5, heart rate zones 1–5; TD, total distance, SZ1-5, speed zones 1–5, Acc1-4, number of accelerations in zones 1–4; Dec1-4, number of decelerations in zones 1–4.

### Internal load variables

**Fig 2:**
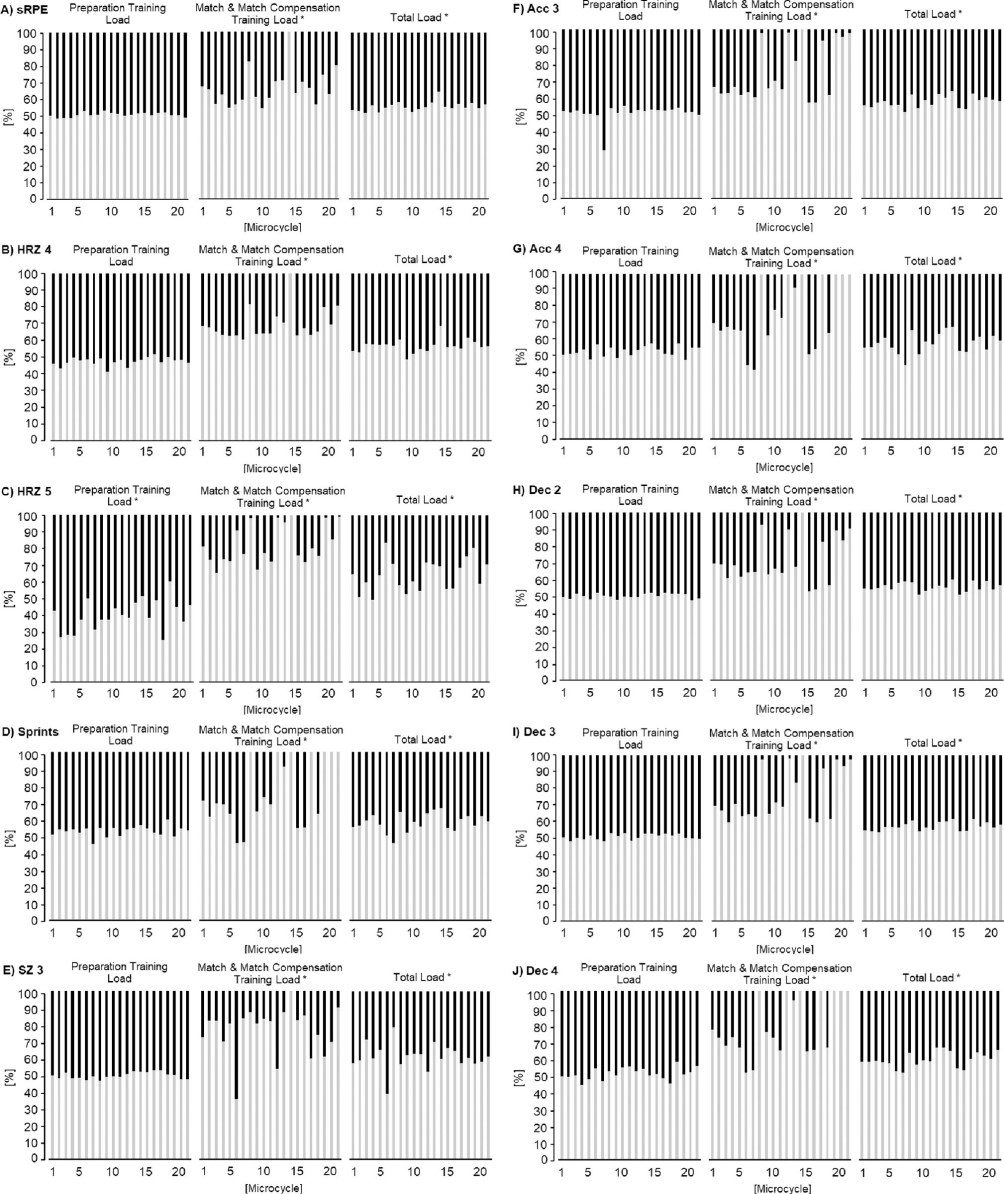
Overview of the distributions of loads for all variables remaining significant in TL between starters (grey) and non-starters (black) (* p < 0.05). A) session rate of perceived exertion; B) time in heart rate zone 4; C) time in heart rate zone 5; D) number of sprints; E) distance in speed zone 3; F) number of accelerations in zone 3; G) number of accelerations in zone 4; H) number of decelerations in zone 2; I) number of decelerations in zone 3; J) number of decelerations in zone 4.

The mean data of all internal variables for PT, M&MCT and TL are summarized in Tables 3–5. The main findings with statistical differences between starters and non-starters for PT were (see Fig 2 and Table 3):

1. Non-starter’s HRZ5 was higher compared to non-starters during PT (+46.1%; *p* < 0.05; d = -0.63)

**Table 3:** Overview of the mean load during PT per week for starters and non-starters over all 21 microcycles.

Variable		Group		Statistical Analysis		
		Starters Mean ± SD (95% CI)	Non-starters Mean ± SD (95% CI)	Cohens d, *interpretation*	*Difference*	*p*-level
	**Internal Load Variables**					
sRPE (AU)		1792.6 ± 814.3 (629.9–2955.2)	1744.0 ± 786.8 (620.7–2867.3)	0.06, *trivial*	-2.7%	0.845
HRZ1 (min)		86.4 ± 30.9 (42.3–130.6)	79.5 ± 34.1 (30.8–128.2)	0.21, *small*	-8.0%	0.496
HRZ2 (min)		88.6 ± 35.1 (38.5–138.8)	92.8 ± 33.7 (44.7–141.0)	-0.12, *trivial*	+4.7%	0.695
HRZ3 (min)		59.5 ± 24.9 (23.9–95.0)	65.3 ± 25.9 (28.4–102.3)	-0.23, *small*	+9.9%	0.459
HRZ4 (min)		39.9 ± 18.6 (13.3–66.4)	44.7 ± 20.7 (15.1–74.2)	-0.24, *small*	+12.0%	0.437
HRZ5 (min)		14.1 ± 9.3 (0.8–27.4)	20.6 ± 11.3 (4.5–36.7)	-0.63, *moderate*	+46.1%	**< 0.05** *
	**External Load Variables**					
TD (m)		21431.2 ± 8071.3 (9907.9–32954.6)	21524.2 ± 8280.3 (9702.3–33346.0)	-0.01, *trivial*	+0.4%	0.971
Sprints (n)		59.9 ± 26.1 (22.6–97.2)	53.2 ± 23.9 (19.1–87.3)	0.27, *small*	-11.2%	0.39
SZ1 (m)		8299.4 ± 3073.5 (3911.4–12687.4)	8279.5 ± 3138.8 (3798.1–12760.8)	0.01, *trivial*	-0.2%	0.984
SZ2 (m)		5361.3 ± 2099.3 (2364.1–8358.4)	5641.4 ± 2305.9 (2349.2–8933.5)	-0.13, *trivial*	+5.2%	0.683
SZ3 (m)		2956.3 ± 1278.2 (1131.4–4781.2)	2975.4 ± 1205.9 (1253.8–4697.0)	-0.02, *trivial*	+0.6%	0.961
SZ4 (m)		1715.1 ± 1490.3 (-412.6–3842.8)	1699.3 ± 1534.4 (-491.4–3890.0)	0.01, *trivial*	-0.9%	0.973
SZ5 (m)		934.7 ± 496.5 (225.9–1643.6)	836.4 ± 455.4 (186.2–1486.6)	0.21, *small*	-10.5%	0.535
Acc1 (n)		1099.6 ± 424.9 (492.9–1706.3)	1094.3 ± 446.5 (456.9–1731.7)	0.01, *trivial*	-0.5%	0.998
Acc2 (n)		606.6 ± 247.8 (252.7–960.4)	607.8 ± 261.0 (235.2–980.3)	-0.01, *trivial*	+0.2%	0.956
Acc3 (n)		174.5 ± 77.3 (64.2–284.8)	162.3 ± 69.5 (63.1–261.5)	0.17, *trivial*	-7.0%	0.667
Acc4 (n)		43.2 ± 19.3 (15.7–70.7)	37.5 ± 17.5 (12.4 62.5)	0.31, *small*	-13.3%	0.43
Dec1 (n)		1294.8 ± 490.1 (595.0–1994.5)	1217.8 ± 470.7 (545.7–1889.9)	0.16, *trivial*	-5.9%	0.677
Dec2 (n)		659.4 ± 266.4 (279.0–1039.8)	655.3 ± 278.8 (257.3–1053.2)	0.02, *trivial*	-0.6%	0.967
Dec3 (n)		166.8 ± 69.1 (68.2–265.4)	158.8 ± 64.2 (67.2–250.4)	0.12, *trivial*	-4.8%	0.767
Dec4 (n)		40.9 ± 18.9 (14.0–67.8)	38.2 ± 17.0 (14.0–62.4)	0.15, *trivial*	-6.6%	0.712

* Different between starters and non-starters (p < 0.05). sRPE, session rate of perceived exertion; HRZ1-5, heart rate zones 1–5; TD, total distance, SZ1-5, speed zones 1–5, Acc1-4, number of accelerations in zones 1–4; Dec1-4, number of decelerations in zones 1–4.

**Table 4:** Overview of the mean load during M&MCT per week for starters and non-starters over all 21 microcycles.

Variable		Group		Statistical Analysis		
		Starters Mean ± SD (95% CI)	Non-starters Mean ± SD (95% CI)	Cohens d, *interpretation*	*Difference*	*p*-level
	**Internal Load Variables**					
sRPE (AU)		1113.6 ± 177.9 (859.6–1367.5)	605.3 ± 284.2 (199.6–1011.0)	2.14, *very large*	-45.6%	**< 0.05** *
HRZ1 (min)		15.3 ± 6.6 (5.8–24.8)	17.6 ± 12.6 (-0.4–35.5)	-0.22, *small*	+14.6%	0.475
HRZ2 (min)		20.0 ± 7.5 (9.2–30.7)	24.3 ± 15.1 (2.7–45.9)	-0.36, *small*	+21.7%	0.246
HRZ3 (min)		24.4 ± 7.6 (13.6–35.2)	19.8 ± 11.6 (3.2–36.6)	0.47, *small*	-18.9%	0.134
HRZ4 (min)		41.4 ± 3.8 (36.0–46.7)	19 ± 7.1 (8.9–29.1)	3.95, *very large*	-54.0%	**< 0.05** *
HRZ5 (min)		34 ± 6.8 (24.3–43.7)	7.5 ± 5.1 (0.2–14.9)	4.39, *extr*. *large*	-77.8%	**< 0.05** *
	**External Load Variables**					
TD (m)		14336.9 ± 2279.5 (11082.4–17591.3)	9033.4 ± 4074.8 (3215.8–14851.0)	1.61, *large*	-37.0%	**< 0.05** *
Sprints (n)		29.7 ± 5.4 (21.9–37.4)	12.4 ± 12.2 (-5.0–29.9)	1.82, *large*	-58.1%	**< 0.05** *
SZ1 (m)		3711.8 ± 791.6 (2581.6–4842.0)	1816.8 ± 1291.2 (-26.7–3660.3)	1.77, *large*	-51.1%	**< 0.05** *
SZ2 (m)		4166.1 ± 1094.3 (2603.7–5728.5)	3963.3 ± 1812.8 (1375.2–6551.4)	0.14, *trivial*	-4.9%	0.663
SZ3 (m)		3770.2 ± 1152.6 (2124.6–5415.8)	1452.2 ± 1396.3 (-541.3–3445.7)	1.81, *large*	-61.5%	**< 0.05** *
SZ4 (m)		1241.1 ± 126.4 (1060.7–1421.5)	499.4 ± 521.9 (-245.7–1244.6)	1.95, *large*	-59.8%	**< 0.05** *
SZ5 (m)		886.1 ± 80.2 (771.6–1000.7)	858.7 ± 345.5 (365.4–1352.0)	0.11, *trivial*	-3.1%	0.725
Acc1 (n)		480.1 ± 74.2 (374.2–586.1)	225.3 ± 155.7 (3.0–447.6)	2.09, *very large*	-53.1%	**< 0.05** *
Acc2 (n)		304.4 ± 65.4 (211.0–397.7)	133.1 ± 81.9 (16.2–250.1)	2.31, *very large*	-56.3%	**< 0.05** *
Acc3 (n)		94 ± 12.8 (75.7–112.3)	38.3 ± 28.0 (-1.6–78.3)	2.56, *very large*	-59.2%	**< 0.05** *
Acc4 (n)		21.2 ± 4.4 (14.9–27.5)	9.0 ± 9.3 (-4.3–22–2)	1.68, *large*	-57.8%	**< 0.05** *
Dec1 (n)		511.0 ± 93.3 (377.8–644.3)	276.4 ± 180.3 (19.0–533.9)	1.63, *large*	-45.9%	**< 0.05** *
Dec2 (n)		326.7 ± 48.2 (257.8–395.5)	146.2 ± 89.6 (18.2–274.2)	2.51, *very large*	-55.2%	**< 0.05** *
Dec3 (n)		86.6 ± 10.1 (72.1–101.1)	31.9 ± 23.2 (-1.3–65.0)	3.06, *very large*	-63.2%	**< 0.05** *
Dec4 (n)		29.4 ± 3.4 (24.6–34.2)	9.5 ± 8.8 (-3.0–22.0)	3.0, *very large*	-67.7%	**< 0.05** *

* Different between starters and non-starters (p < 0.05). sRPE, session rate of perceived exertion; HRZ1-5, heart rate zones 1–5; TD, total distance, SZ1-5, speed zones 1–5, Acc1-4, number of accelerations in zones 1–4; Dec1-4, number of decelerations in zones 1–4.

**Table 5:** Overview of the mean TL per week for starters and non-starters over all 21 microcycles.

Variable		Group		Statistical Analysis		
		Starters Mean ± SD (95% CI)	Non-starters Mean ± SD (95% CI)	Cohens d, *interpretation*	*Difference*	*p*-level
	**Internal Load Variables**					
sRPE (AU)		2906.1 ± 836.6 (1711.7–4100.6)	2349.3 ± 775.6 (1242.0–3456.6)	0.69, *moderate*	-19.2%	**< 0.05** *
HRZ1 (min)		101.8 ± 32.6 (55.2–148.3)	97.1 ± 36.7 (44.8–149.4)	0.13, *trivial*	-4.6%	0.665
HRZ2 (min)		108.6 ± 35.6 (57.7–159.5)	117.1 ± 34.5 (67.9–166.4)	-0.24, *small*	+7.9%	0.435
HRZ3 (min)		83.9 ± 25.2 (47.9–119.8)	85.1 ± 25.8 (48.3–121.9)	-0.05, *trivial*	+1.5%	0.875
HRZ4 (min)		81.3 ± 19.5 (53.5–109.0)	63.7 ± 20.3 (34.8–92.6)	0.88, *moderate*	-21.6%	**< 0.05** *
HRZ5 (min)		48.1 ± 10.8 (32.6–63.5)	28.1 ± 11.5 (11.7–44.6)	1.78, *large*	-41.4%	**< 0.05** *
	**External Load Variables**					
TD (m)		35768.1 ± 8342.0 (23858.2–47678.0)	30557.5 ± 8521.4 (18391.6–42723.5)	0.62, *moderate*	-14.6%	0.052
Sprints (n)		89.6 ± 27.0 (51.1–128.1)	65.5 ± 24.7 (30.3–100.9)	0.93, *moderate*	-26.7%	**< 0.05** *
SZ1 (m)		12011.2 ± 3173.3 (7480.8–16541.7)	10096.3 ± 3198.1 (5530.4–14662.2)	0.60, *moderate*	-15.9%	0.058
SZ2 (m)		9527.4 ± 2329.9 (6201.0–12853.7)	9604.7 ± 2636.3 (5840.8–13368.5)	-0.03, *trivial*	+0.8%	0.92
SZ3 (m)		6726.5 ± 1754.6 (4221.4–9231.6)	4427.6 ± 1605.7 (2135.1–6720.1)	1.37, *large*	-34.2%	**< 0.05** *
SZ4 (m)		2956.1 ± 1483.3 (848.4–5073.8)	2198.8 ± 1620.3 (-114.6–4512.1)	0.49, *small*	-25.6%	0.122
SZ5 (m)		1820.8 ± 492.4 (1117.8–2523.8)	1702.4 ± 600.4 (845.2–2559.6)	0.22, *small*	-6.5%	0.489
Acc1 (n)		1579.8 ± 447 (941.6–2217.9)	1324.5 ± 451.6 (679.8–1969.2)	0.57, *small*	-16.2%	0.073
Acc2 (n)		911.0 ± 274.6 (518.9–1303.1)	744 ± 262.2 (369.7–1118.3)	0.62, *moderate*	-18.3%	0.051
Acc3 (n)		268.5 ± 79.8 (154.6–382.4)	203.1 ± 70.3 (102.8–303.4)	0.87, *moderate*	-24.4%	**< 0.05** *
Acc4 (n)		64.4 ± 20.1 (35.6–93.1)	47.6 ± 18.7 (20.8–74.3)	0.86, *moderate*	-26.1%	**< 0.05** *
Dec1 (n)		1805.8 ± 518.7 (1065.3–2546.3)	1509.3 ± 477.6 (827.5–2191.2)	0.59, *small*	-16.4%	0.061
Dec2 (n)		986.0 ± 282.3 (583.0–1389.1)	802.1 ± 285.0 (395.2–1209.1)	0.65, *moderate*	-18.7%	**< 0.05** *
Dec3 (n)		253.4 ± 71.5 (151.4–355.4)	192.5 ± 63.0 (102.5–282.5)	0.9, *moderate*	-24.0%	**< 0.05** *
Dec4 (n)		70.3 ± 19.5 (42.4–98.2)	48.3 ± 15.3 (26.5–70.2)	1.25, *large*	-31.2%	**< 0.05** *

* Different between starters and non-starters (p < 0.05). sRPE, session rate of perceived exertion; HRZ1-5, heart rate zones 1–5; TD, total distance, SZ1-5, speed zones 1–5, Acc1-4, number of accelerations in zones 1–4; Dec1-4, number of decelerations in zones 1–4.

The main findings with statistical differences between starters and non-starters for M&MCT were (see Fig 2 and Table 4):

1. Lower values in sRPE were found for non-starters compared to starters (-45.6%; *p* < 0,05; d = 2.14)
2. Compared to starters, non-starters spent less time in HRZ4 (-54%; *p* < 0.05; d = 3.95) and HRZ5 (-77.8%; *p* < 0.05; d = 4.39)

The main findings with statistical differences between starters and non-starters for TL were (see Fig 2 and Table 5):

1. sRPE remained decreased for non-starters compared to starters (-19.2%; *p* < 0.05; d = 0.69)
2. Lower values for non-starters compared to starters were found in HRZ4 (-21.6%; *p* < 0.05; d = 0.88) and HRZ5 (-41.4%; *p* < 0.05; d = 1.78)

Comparative analyses between starters and non-starters across the remaining internal load variables yielded no statistically significant differences (Tables 3–5; all *p* > 0.05).

### External load variables

All external variables for PT, M&MCT and TL are summarized in Tables 3–5. No statistical differences between starters and non-starters were found in PT (see Table 3).

The main findings with statistical differences between starters and non-starters for M&MCT were (see Fig 2 and Table 4):

1. Lower values were found in TD for non-starters compared to starters (-37%; *p* < 0.05; d = 1.61)
2. Non-starters showed a lower number of sprints compared to starters (-58.1%; *p* < 0.05; d = 1.82)
3. Comparing the values of running distance in different speed zones, lower values could be found for non-starters compared to starters in SZ1 (-51.1%; *p* < 0.05; d = 1.77), SZ3 (-61.5%; *p* < 0.05; d = 1.81) and SZ4 (-59.8%; *p* < 0.05; d = 1.95)
4. Regarding the number of accelerations, all zones were decreased for non-starters compared to starters (Acc1: -53.1%; *p* < 0.05; d = 2.09; Acc2: -56.3%; *p* < 0.05; d = 2.31; Acc3: -59.2%; *p* < 0.05; d = 2.56; Acc4: -57.8%; *p* < 0.05; d = 1.68)
5. Regarding the number of decelerations, all zones were decreased for non-starters compared to starters (Dec1: -45.9%; *p* < 0.05; d = 1.63; Dec2: -55.2%; *p* < 0.05; d = 2.51; Dec3: -63.2%; *p* < 0.05; d = 3.06; Dec4: -67.7%; *p* < 0.05; d = 3).

The main findings with statistical differences between starters and non-starters for TL were (see Fig 2 and Table 5):

1. Non-starters showed lower number of sprints compared to starters (-26.7%; *p* < 0.05; d = 0.93)
2. Values in speed zones remained decreased for non-starters compared to starters in SZ3 (-34.2%; *p* < 0.05; d = 1.37)
3. The number of accelerations remain decreased for non-starters compared to starters in Acc3 (-24.4%; *p* < 0.05; d = 0.87) and Acc4 (-26.1%; *p* < 0.05; d = 0.68)
4. The number of decelerations remain decreased for non-starters compared to starters in Dec2 (-18.7%; *p* < 0.05; d = 0.65), Dec3 (-24%; *p* < 0.05; d = 0.9) and Dec4 (-31.2%; *p* < 0.05; d = 1.25)

Comparative analyses between starters and non-starters across the remaining external load variables yielded no statistically significant differences (Tables 3–5; all *p* > 0.05).

## Discussion

The major findings concerning internal load variables were as follows:

1. sRPE was similar during PT for starters and non-starters, however during M&MCT and for TL the non-starters showed lower values.
2. No differences in HRZ1, HRZ2 and HRZ3 for all parts of the microcycles (PT, M&MCT and TL) were found between the groups.
3. For HRZ4, only during PT values were similar between starters and non-starters, during M&MCT and for TL higher values were found for starters compared to non-starters.
4. For HRZ5, non-starters showed higher values during PT. During M&MCT and for TL, values were higher for the starters vs non-starters.

The major findings concerning external load variables were as follows:

1. All SZ (1–5) were similar during PT.
2. During M&MCT higher values were found for starters in SZ1, SZ3 and SZ4.
3. For TL increased load for starters was only calculated in SZ3.
4. All Acc (1–4) and Dec (1–4) were similar between the groups during PT.
5. All Acc (1–4) and Dec (1–4) showed increased values for starters during M&MCT.
6. Higher values in Acc (1–4) and Dec (1–4) for TL were calculated in Acc3, Acc4, Dec2, Dec3 and Dec4. All other SZ (Acc1, Acc2 and Dec1) were similar in TL.

In the present study, 10 players accounted for 82.1% of the data for the group of starters, while another 10 players were never allocated to this group over the 21 weeks. This highlights the practical relevance of addressing the issue of uneven load distributions in official matches. When a small number of players contribute to a significant portion of the overall match loads, it can lead to an increased risk of injury for those players [16]. Simultaneously, diminished fitness for the rest of the team could be a consequence, as official matches are crucial for maintaining and improving player performance [34].

### Internal load variables

Since matches represent the highest training peaks of the microcycle for both internal and external loads [35–37], missing these loads as a non-starter can result in significantly decreased stresses that accumulate over the weeks. Overall, the time spent in HRZ5 during M&MCT was 26.5 min longer for the starters compared to the non-starters attributable to the differential high-intensity loading time between the two cohorts. During standard competitive matches, players generally manifest an internal load corresponding to approximately 85% of their HR_max_ [12, 38] over 90 min (exclusive additional playing time). In contrast SSGs, contingent upon various parameters such as player count and field dimensions, induce elevated mean values ranging from 85% to 91% of HR_max_ [8, 9, 39], comparable in extent to the values achieved in matches. However, it is noteworthy that the duration of SSGs is typically confined to 8–16 minutes, as opposed to the 90-minute duration of a standard match. Adding the time spent in HRZ4 (> 80% HR_max_), the accumulated difference in high intensity exposure (i.e. HRZ4 and HRZ5) sums up to approx. 42 min per week between starters and non-starters for TL.

The present data is in line with previous findings [10] showing a large time of a match is spent in HRZ 3–5 (65%). Running-based sessions on MD, as well as immediate post-match runs for players with limited playing time (< 45 minutes), and MCT appear to adequately compensate for the reduced duration in HRZ3 for non-starters. This is in alignment with previous findings that have reported significantly lower durations spent above 90% of HR_max_ for non-starters in microcycles that include matches [13].

Interestingly, despite the homogeneity in training session design and loading parameters for both starters and non-starters during PT, no significant disparities were observed in all internal and external variables, with the exception of HRZ5. In this specific zone, non-starters exhibited an extended duration of 6.5 minutes compared to starters.

Several factors could account for the elevated time spent in HRZ5 by non-starters. It is well-established that high-intensity efforts contribute to significant adaptations in oxygen uptake, transport, and utilization. High-Intensity Interval Training (HIIT), in particular, is known to facilitate cardiac remodeling, including an increase in stroke volume [40], which subsequently results in a reduced HR response to a given external load. Given that the external load during PT was consistent across both groups, the protracted duration in HRZ5 for starters may serve as an indicator of a less adapted cardiorespiratory system in non-starters relative to starters. Conversely, it is widely acknowledged that peak HR can experience a chronic decrement in response to sustained fatigue [41]. Regrettably, the current study did not encompass the evaluation of fatigue markers such as heart rate variability [42], countermovement jump performance, or biochemical indicators [43], thereby precluding an assessment of individual player fatigue levels.

Nevertheless, although not reaching statistical significance, the external load metrics reveal that starters executed a greater number of sprints, covered more distance in SZ4 and SZ5, and exhibited increased counts of accelerations and decelerations in the higher-intensity zones (Acc 3, Acc 4, Dec 3, and Dec 4) during PT as compared to non-starters. These observations suggest that starters may have exerted a higher level of external effort while manifesting a reduced internal response in HRZ5.

In the current investigation, starters exhibited elevated sRPE values in comparison to non-starters during M&MCT and TL, a result that aligns with prior research [44, 45]. The elevated sRPE values are likely attributable to an extended duration of high-intensity exposure, specifically increased time spent in HRZ4 and HRZ5, during competitive matches as opposed to compensatory training sessions.

### External load variables

Overall, no significant disparities were observed between starters and non-starters across all external load variables during PT. This outcome is attributable to the standardized external load parameters, which were defined at the group level but individually monitored by the athletic coaching staff to align with the team’s overarching loading objectives.

Although not reaching statistical significance, starters exhibited marginally elevated values across all high-intensity external parameters (number of Sprints, SZ4, SZ5, Acc3, Acc4, Dec3 and Dec4), while concurrently displaying significantly reduced time in HRZ5 during PT (as previously discussed; refer to Table 3). The underlying rationale for these observed differences remains elusive. However, as previously discussed, the diminished time allocation in HRZ4 and HRZ5 (cumulatively approximating 42 minutes per week) and potentially lower fitness levels may account for the relatively lower external load metrics and elevated internal load values among non-starters compared to starters.

Furthermore, during M&MCT, distances covered in SZ1, SZ3 and SZ4 were notably greater for starters relative to non-starters. Several factors elucidate this observation: i) a substantial proportion of a soccer match is typically allocated to lower speed zones, contingent upon the specific zonal demarcation [22, 46]. ii) The running-based training regimen for non-starters on MD predominantly consists of activities in SZ2 and SZ5, as summarized in Table 2. This accounts for the comparable distances covered in SZ2 and SZ5 by both starters and non-starters during M&MCT, as indicated in Table 4. While it appears that the MD running-based training effectively compensates for SZ2 and SZ5, it does not yield similar results for TD, SZ1, SZ3, and SZ4.

During MCT on the day following the match (MD+1), soccer-specific drills, including SSGs, are typically incorporated into a training session. The inherent constraints of SSGs, particularly when conducted on smaller pitch dimensions, preclude the stimulation of extended distances or higher-speed zone activities.

As with the HR extent that is comparable to match demands (as previously discussed), SSGs also elicit intensities that reach different speed zones and TD in meters per minute similar to, or sometimes even exceeding, official match play [47]. Again, the shorter net playing time seems to play a deciding role, elucidating the observed reduction in TD, SZ1, SZ3, and SZ4 for non-starters relative to starters. Similar findings of reduced total distance in non-starters have been corroborated in other analyses [47, 48].

In the current investigation, elevated counts of accelerations and decelerations across all specified zones (Acc1-4 and Dec1-4) were observed for starters relative to non-starters during M&MCT. While SSGs, the principal component of MCT, inherently induce frequent directional changes [49] and corresponding alterations in velocity—particularly with decreased pitch sizes [50]—the accumulated number of accelerations and decelerations exhibited by starters during competitive matches could not be replicated for non-starters. This observation is corroborated by prior research indicating that an elevated rate of accelerations per minute in SSGs failed to approximate the absolute count of accelerations observed in friendly matches [20].

In general, TD, running distance in higher speed zones and maximum accelerations and decelerations are elicited in greater extend with increasing pitch dimensions during SSGs [50]. In the MCT setting, the number of players is often limited, which tends to lead to smaller forms of SSG (i.e. 4 vs 4).

Therefore, TD, Acc3, Acc4, Dec2, Dec3 and Dec4 remained diminished for non-starters compared to starters, likely due to their restricted total playing time in SSGs, while comparable counts in Acc1, Acc2 and Dec1 between starters and non-starters within TL imply the additional limitation of SSGs: the constrained pitch dimensions may result in reduced velocities, and consequently, lower levels of accelerations and decelerations (measured in m/s), failing to reach the upper acceleration and deceleration zones (Acc3-4 and Dec2-4).

In anticipation of the forthcoming match, it is imperative that both starting and reserve players on the soccer team undergo equivalent physical training regimens during PT. Often, the time windows of Match Day (MD) and the subsequent days (MD+1, MD+2), largely focused on recompensation for starters [51], present the only opportunities to administer additional training stimuli to players with limited in-game exposure. The objective during this specific temporal interval–MD, MD+1 and MD+2—is to emulate, as closely as possible, the physiological and biomechanical demands encountered during competitive matches [23]. This includes achieving target metrics in elevated heart rate zones, cumulative distance covered, frequency of high-velocity sprints, time spent in elevated speed zones, and the quantity of accelerative and decelerative movements.

Considering the current empirical evidence and the unique contextual factors affecting the team, it is observed that modified MCT with an emphasis on SSGs fails to adequately offset the deficit in match load exposure. Specifically, the inherent limitations of SSGs—such as confined playing areas and reduced duration of play—should be critically evaluated. These limitations result in notably diminished time spent in heart rate zones 4 and 5 (HRZ4 and HRZ5), a reduced number of high-velocity sprints, and less distance covered in elevated speed zones for non-starting players compared to their starting counterparts. While SSGs can approximate the density of accelerative and decelerative movements, they fall short in replicating the aggregate number of such movements observed in a standard 90-minute match.

### Practical considerations

The integration of supplementary running sessions on MD, MD+1, or MD+2, which include exposure to TD, higher-speed zones (SZ3-5), and elevated HR zones (HRZ4-5), may serve as an effective approach to mitigate load discrepancies before focusing on soccer-specific movements through SSGs in MCT. Additionally, the implementation of maximum sprints can address the issue of limited pitch sizes in SSGs during MCT, thereby compensating for locomotive deficits in non-starters. Furthermore, organizing friendly matches or participation in an Under-21 team (if applicable) can maintain sufficient levels of external and internal load exposure for non-starters [23].

### Strength and limitations

The major strength of this investigation include: i) a comprehensive analysis encompassing both internal and external load variables, ii) an extended observational period spanning 21 weeks and iii) the generation of empirically-informed, practical recommendations that hold significant utility for coaching staff.

Conversely, the primary limitations of this study lie in its retrospective nature, focusing solely on a single team within a specific temporal framework. As such, the findings are not generalizable, precluding any definitive conclusions regarding the management of disparate internal and external loads between starting and reserve players in other athletic organizations. In addition, there was no monitoring of individual sessions outside of the training routine with the team, which may have a minor influence on the stress on individual players. Such tracking should be implemented in future research.

Future research in this area would provide valuable insights into how other teams address the issue of unequal distribution of match loads. Additionally, subsequent investigations could explore the effectiveness of specific strategies, derived from the present findings, to balance loads within a microcycle.

## Conclusions

In conclusion, our study reveals significant disparities in internal and external workloads between starting and non-starting professional soccer players over a 21-week period. Non-starters exhibited lower training loads during M&MCT across several key metrics, including sRPE, HRZ, TD, and high-intensity activities. Although the workload during PT was similar, the cumulative weekly load remained unbalanced, particularly in terms of sRPE, HRZ4-5, number of sprints, SZ3, and acceleration/deceleration (Acc3-4, Dec2-4). To address these discrepancies, we recommend implementing additional running-based sessions and modifying SSGs for non-starters on match days and subsequent days. This approach aims to replicate the physiological and biomechanical demands experienced by starters, thereby supporting the physical development and preparedness of non-starters.
